# Human dystrophin tandem calponin homology actin-binding domain crystallized in a closed-state conformation

**DOI:** 10.1107/S2059798325001457

**Published:** 2025-02-26

**Authors:** Oakley Streeter, Ke Shi, Joseph Vavra, Hideki Aihara, James M. Ervasti, Robert Evans, Joseph M. Muretta

**Affiliations:** a Department of Biochemistry, Molecular Biology and Biophysics University of Minnesota Minneapolis MN55455 USA; McGill University, Canada

**Keywords:** actin binding, dystrophin, DMD, cytoskeleton, calponin homology

## Abstract

The structure of the N-terminal actin-binding domain of human dystrophin was determined at 1.94 Å resolution. Each chain in the asymmetric unit exists in a ‘closed’ conformation, with the first and second calponin homology (CH) domains directly interacting via a 2500.6 Å^2^ interface. The positioning of the individual CH domains is comparable to the domain-swapped dimer seen in previous human dystrophin and utrophin actin-binding domain 1 structures. The CH1 domain is highly similar to the actin-bound utrophin structure and structural homology suggests that the ‘closed’ single-chain conformation opens during actin binding to mitigate steric clashes between CH2 and actin.

## Abstract 2

The structure of the N-terminal actin-binding domain of human dystrophin was determined, revealing a closed conformation with the first and second calponin homology domains directly interacting.

## 1. Introduction

Dystrophin is a cytoskeletal protein belonging to the spectrin superfamily and is responsible for linking the extracellular matrix (ECM)-binding dystroglycan complex to the cortical actin cytoskeleton (Ervasti & Campbell, 1993 ▸). Mutations in the dystrophin gene cause diseases including Becker and Duchenne muscular dystrophy, in part by disrupting the cytoskeletal–dystroglycan–ECM linkage. Dystrophin binds dystroglycan via its C-terminal cysteine-rich dystroglycan-binding domain. Dystroglycan in turn binds the extracellular matrix outside cells. Actin binding by dystrophin is mediated by two portions of the protein: the tandem calponin homology (CH) actin-binding domain 1 (ABD1, residues 1–246) and a series of spectrin repeats (SR) in the middle of the protein comprised of SR11–SR17 and referred to as actin-binding domain 2 (ABD2; residues Phe1461–Gln2209) (Ervasti, 2007 ▸; Rybakova *et al.*, 1996 ▸, 2006 ▸; Amann *et al.*, 1998 ▸). Biochemical studies investigating isolated dystrophin ABD1 suggest that it binds actin with a micromolar affinity, ranging from 10 to >60 µ*M* in published reports (Rybakova *et al.*, 2006 ▸; Bañuelos *et al.*, 1998 ▸; Renley *et al.*, 1998 ▸; Way *et al.*, 1992 ▸; Upadhyay *et al.*, 2020 ▸), compared with full-length dystrophin, which binds filamentous actin (F-actin) with nanomolar affinity (Rybakova *et al.*, 1996 ▸, 2006 ▸).

The ABD1 of dystrophin is a member of the calponin homology domain superfamily. CH domains are found in a range of actin-binding proteins (Bañuelos *et al.*, 1998 ▸; Norwood *et al.*, 2000 ▸) and consist of approximately 100 amino-acid residues with a characteristic α-helical fold that includes a core three-helix bundle and two flanking helices. CH domains were first characterized in calponin, an actin-binding protein that regulates the interaction between smooth muscle myosin and actin thin filaments in smooth muscle (Yin *et al.*, 2020 ▸). Proteins containing a single N-terminal CH domain include calponin, as well as various signaling proteins. Proteins with an actin-binding domain (ABD) that contain two CH domains in tandem include dystrophin, utrophin, α-actinin, β-spectrin, filamin and plectin. The plastin/fimbrin family contains two tandem CH domains in series (Korenbaum & Rivero, 2002 ▸; Gimona *et al.*, 2002 ▸).

The tandem CH ABDs of α-actinin, β-spectrin, filamin and plectin have crystal structures in a monomeric ‘closed’ conformation with the first (CH1) and second (CH2) domains interacting across a 1500–3000 Å^2^ interface (Norwood *et al.*, 2000 ▸; Djinovic Carugo *et al.*, 1997 ▸; Sawyer *et al.*, 2009 ▸; García-Alvarez *et al.*, 2003 ▸; Goldsmith *et al.*, 1997 ▸). The crystal structures of dystrophin (PDB entry 1dxx) and utrophin (PDB entry 1qag) differ in their conformation. In both, single chains form an antiparallel domain-swapped dimer, with the CH domains of each monomer extended in an ‘open’ conformation but interacting with the opposing CH domain of a second chain (Norwood *et al.*, 2000 ▸; Keep *et al.*, 1999 ▸) via interfaces of 2327.5 and 2182.5 Å^2^, respectively. The domain-swapped CH1 and CH2 domains in these structures interact via an interface that is highly similar to the CH1–CH2 interface seen in the closed-state structures of α-actinin, β-spectrin, filamin and plectin. This suggests that a monomeric closed conformation of the dystrophin ABD1 forms in solution (Borrego-Diaz *et al.*, 2006 ▸). Spectroscopic and modeling studies support the hypothesis that the dystrophin and utrophin N-terminal ABD domains transition between open and closed conformations. The structure and physiological importance of these states is not understood and neither is their impact on actin binding. To investigate this, we crystallized human dystrophin ABD1 in a monomeric closed conformation that does not exhibit the domain-swapped configuration seen in previous dystrophin (PDB entry 1dxx) or utrophin (PDB entry 1qag) structures.

## 2. Methods

### 2.1. Cloning

Wild-type and Cys-Lite human dystrophin ABD1 (hDys-ABD1) expression plasmids were constructed by Gibson assembly of synthetic Gene Blocks (Gibson *et al.*, 2009 ▸). The DNA sequence for dystrophin residues 2–246 (WT-ABD1), corresponding to ABD1 in accession No. NM_004006.3, and residues 2–246 with C10S and C188S amino-acid substitutions (Cys-Lite-ABD1) were synthesized with 20 bp 5′ and 3′ extensions homologous to the expression plasmid by Integrated DNA Technologies. The expression plasmid, pTD68, is a pET plasmid containing an N-terminal 6×His-SUMO tag followed by a multiple cloning site prior to the terminator (Aird *et al.*, 2018 ▸). The parent vector was engineered with AgeI and XhoI cleavage sites between the BamHI site 3′ to the SUMO tag and the T7 terminator and linearized by restriction digestion using AgeI and XhoI (New England Biolabs) followed by insertion of the Gene Block using Gibson Assembly Master Mix (New England Biolabs). The assembled plasmid was transformed into competent *Escherichia coli* DH5α cells and plated onto 100 µg ml^−1^ ampicillin plates. Plasmids were purified from positive transformants and then sequence-verified by Sanger sequencing.

### 2.2. Protein expression and purification

**Figure 1:**
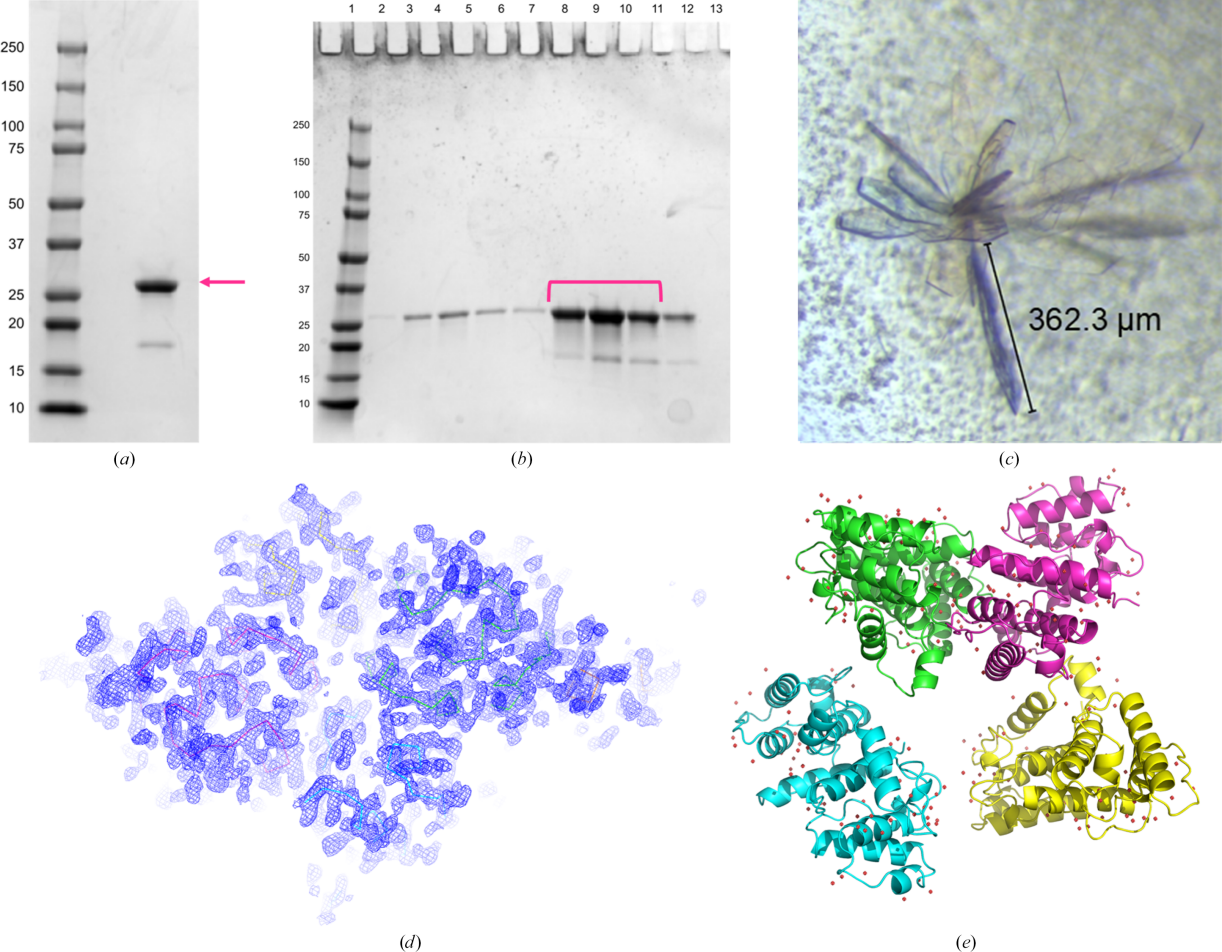
Crystallization and modeling of hDys-C10S-C188S-ABD1. (*a*) SDS–PAGE gel stained with Coomassie staining showing representative recombinant hDys-C10S-C188S-ABD1 used for crystallization (magenta arrow, 3 µg of protein loaded, molecular weight 28.6 kDa). (*b*) Size-exclusion chromatography of hDys-C10S-C188S-ABD1. The magenta brackets indicate the fractions pooled to obtain the material in (*a*). (*c*) Representative crystal of hDys-C10S-C188S-ABD1. (*d*) Molecular replacement with the *AlphaFold*2 model of hDys-C10S-C188S-ABD1 residues 2–246 showing the backbone (lines) and electron density (2*F*_o_ − *F*_c_ map contoured at 1.5σ). (*e*) Asymmetric unit of the final refined model: chains *A*, *B*, *C* and *D* are shown in green, cyan, magenta and yellow, respectively. Crystallographic waters are shown as red dots.

Verified plasmids were transformed into *E. coli* NiCo21 (DE3) cells (New England Biolabs) and cultured in 1 l Luria–Bertani (LB) broth with 100 µg ml^−1^ ampicillin at 37°C. The culture was induced at an OD_600_ of between 0.6 and 0.9 using 1 m*M* isopropyl β-d-1-thiogalactopyranoside (IPTG) and the induced cells were grown for 18 h at 18°C. The cells were harvested by centrifugation and the pellet was resuspended in lysis buffer (400 m*M* NaCl, 7.8 m*M* KCl, 10 m*M* Na_2_HPO_4_, 1.8 m*M* KH_2_PO_4_ pH 7.5, 10 m*M* imidazole). A cOmplete EDTA-free protease-inhibitor tablet, Pefabloc SC and DNase I (Roche) were added as per the manufacturer’s specifications to prevent degradation and minimize DNA contamination. Lysis was performed via sonication at 4°C using a Branson Sonifier 450 set to 50% duty cycle for 30 s pulse intervals totaling 10 min. The homogenous suspension was then centrifuged at 41 060*g* for 30 min at 4°C. The resulting supernatant was flowed over a lysis buffer-equilibrated Qiagen 5 ml Ni–NTA Superflow cartridge followed by 100 ml wash buffer (400 m*M* NaCl, 7.8 m*M* KCl, 10 m*M* Na_2_HPO_4_, 1.8 m*M* KH_2_PO_4_ pH 7.5, 25 m*M* imidazole) and 30 ml elution buffer (400 m*M* NaCl, 7.8 m*M* KCl, 10 m*M* Na_2_HPO_4_, 1.8 m*M* KH_2_PO_4_ pH 7.5, 300 m*M* imidazole). Elution fractions (3 ml) were evaluated for protein content by mixing 10 µl sample with 100 µl Bradford reagent (Bio-Rad), inspected for relative blue appearance and pooled. Dithiothreitol (DTT) was added to the pooled fractions to a concentration of 1 m*M* and 400 µl of the SUMO protease ULP1 (1 mol per 3 mol purified SUMO-ABD1 protein) was added to cleave the tag from the N-terminus of ABD1 (Fig. 1 ▸). The sample was then dialyzed overnight in dialysis buffer 1 (400 m*M* NaCl, 7.8 m*M* KCl, 10 m*M* Na_2_HPO_4_, 1.8 m*M* KH_2_PO_4_ pH 7.5, 1 m*M* DTT) followed by dialysis into dialysis buffer 2 (400 m*M* NaCl, 7.8 m*M* KCl, 10 m*M* Na_2_HPO_4_, 1.8 m*M* KH_2_PO_4_ pH 7.5) with 50 m*M* imidazole and no DTT for 2 h. The resulting sample was incubated for 30 min with HisPur Ni–NTA resin (Thermo Fisher) to bind the cleaved 6×His-SUMO tag and centrifuged in spin columns to remove the resin, SUMO tag and ULP1. The flowthrough was collected and further purified using a Superdex 75 10/300 GL Increase size-exclusion chromatography column (Cytiva) while also undergoing buffer exchange into 20 m*M* HEPES, 200 m*M* NaCl, 1 m*M* TCEP. Fractions containing the 28.6 kDa target protein (Fig. 1 ▸) were pooled, concentrated to 9.48 mg ml^−1^ using a spin concentrator (Amicon Ultra-0.5 Centrifugal Filter Unit, 3 kDa molecular-weight cutoff) and then used for crystallization studies.

### 2.3. Crystallization

Protein samples were subjected to crystallization screening at 9.48 mg ml^−1^ over a broad range of common conditions. The most promising condition produced crystals of the Cys-Lite-ABD1 sample within one day. Consistent with previous studies, WT-ABD1 containing native cysteines did not crystallize under any of the >1000 conditions tested. Conditions that gave notable crystallization in the screen of Cys-Lite-ABD1 were optimized by hanging-drop vapor diffusion. Three droplet volume ratios (1.6:1.2 µl, 1.2:1.6 µl and 1:1 µl protein solution:reservoir solution) consisting of protein solution at 9.2 mg ml^−1^ and reservoir solution were set up on 22 mm siliconized cover slides and sealed on a 24-well plate. The reservoir solutions were composed of 0.1 *M* bis-Tris and polyethylene monomethyl ether (MPEG) 5000 covering a range of pH values (6.5–7.5 in 0.2-unit increments) and MPEG concentrations (10–30% in 5% increments). Crystals that formed from the 0.1 *M* bis-Tris pH 6.7, 30%(*w*/*v*) MPEG 5000 condition were harvested and made into micro-seed stocks with a Seed Bead kit (Hampton Research) that were used to seed Cys-Lite-ABD1 by adding 0.5 µl 1:100 dilution seed stock on top of previously described droplet ratios (D’Arcy *et al.*, 2014 ▸; Luft & DeTitta, 1999 ▸). The seeded Cys-Lite-ABD1 sample crystallized readily. Crystals formed at 18°C within one day. We harvested representative crystals (see, for example, Fig. 1 ▸) in 25% ethylene glycol and shipped them to SSRL beamline 12-2 for data collection.

### 2.4. Data processing, refinement and analysis

The X-ray diffraction data set used for refinement is summarized in Table 1 ▸. Data were acquired under cryo-conditions on SSRL beamline 12-2 and NSLS2 beamline AMX with a wavelength of 0.979 Å using EIGER 9M and EIGER 16M pixel-array detectors (PADs). We performed initial data processing and error modeling using *HKL*-2000 (Otwinowski & Minor, 1997 ▸), cutting off the resolution at 1.94 Å with an *I*/σ(*I*) of 2.1. Structure solution and refinement were carried out using computational resources at the Minnesota Supercomputing Institute (MSI). *AlphaFold*2 (Jumper *et al.*, 2021 ▸) implemented at MSI was used to obtain a molecular-replacement model for the crystallized protein sequence. The replacement model was trimmed using *PyMOL* (version 2.5; Schrödinger), removing residues with low *AlphaFold* pLDDT values. The trimmed regions included residues 1–19 at the N-terminus of the protein, residues 241–248 at the C-terminus and residues 126–139 in the linker between the CH domains. The structure was solved with *Phaser* (McCoy *et al.*, 2007 ▸) using the trimmed *AlphaFold*2-predicted model and was refined with *Phenix* 1.20.1-4487 (Liebschner *et al.*, 2019 ▸) and *Coot* (Emsley *et al.*, 2010 ▸). *MolProbity* (Chen *et al.*, 2010 ▸) was used for Ramachandran analysis. Initial crystal refinement reached an *R*_work_ value of 0.2853 and an *R*_free_ value of 0.3393, which did not decrease with further refinement until a twin-law parameter was included, decreasing *R*_work_ to 0.2239 and *R*_free_ to 0.2727. *Phenix.xtriage* was used to evaluate the data set for twinning and generate a suitable twin law. Interactions between the CH1 and CH2 domains were analyzed using *PyMOL* and *ChimeraX* (Pettersen *et al.*, 2021 ▸).

## 3. Results and discussion

### 3.1. Crystallization and structure determination

**Figure 2:**
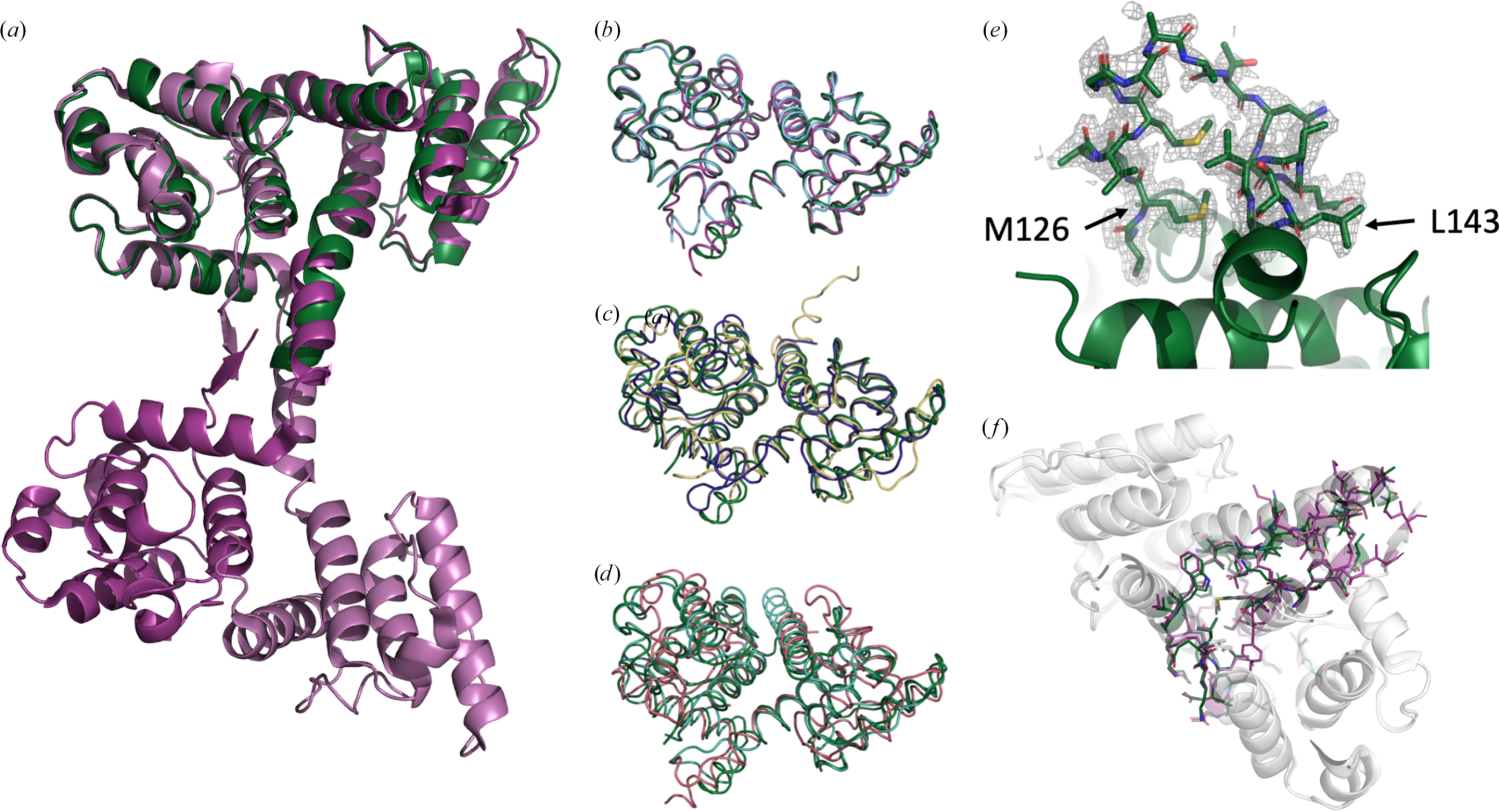
Backbone homology. (*a*) Alignment of PDB entry 9d58 chain *A* (green) with PDB entry 1dxx chains *A* (pink) and chain *B* (violet). Backbone homology is shown between PDB entry 9d58 (green) and (*b*) the CH1 and CH2 domains of domain-swapped dystrophin ABD1 (PDB entry 1dxx, violet) and utrophin (PDB entry 1qag, light blue), (*c*) α-actinin (PDB entry 2eyi, purple) and filamin (PDB entry 2wa5, yellow) and (*d*) fimbrin (PDB entry 1aoa, brick) and plectin (PDB entry 1mb8, cyan). (*e*) Electron density in PDB entry 9d58 for residues 125–144 of chain *A* connecting the CH1 and CH2 domains (2*F*_o_ − *F*_c_ map contoured at 1.5σ). (*f*) Interacting residues (PDB entry 9d58 in green, PDB entry 1dxx in violet) in the interface between the CH1 and CH2 domains.

The Cys-Lite-ABD1 crystals (Fig. 1 ▸) belonged to space group *P*12_1_1. The unit-cell parameters were *a* = 59.77, *b* = 143.13, *c* = 59.86 Å, α = 90.00, β = 102.21, γ = 90.00°. There were four protein molecules in the asymmetric unit. The Matthews coefficient was 2.24 Å^3^ Da^−1^ and the solvent fraction was 45.2%. Following data acquisition and initial data processing, *AlphaFold*2 was used to generate models of hDys-ABD1 (residues 2–246), hDys-CH1 (residues 2–130) and hDys-CH2 (residues 131–248). The models were used for molecular replacement (Fig. 1 ▸). The *AlphaFold*2 hDys-ABD1 model placed the CH domains in a ‘closed’ conformation, similar to the closed conformations observed in the crystal structures of other tandem CH domains such as α-actinin 1 (PDB entry 2eyi), filamin B (PDB entry 2wa5), fimbrin (PDB entry 1aoa) and plectin (PDB entry 1mb8) (Borrego-Diaz *et al.*, 2006 ▸; Sawyer *et al.*, 2009 ▸; Goldsmith *et al.*, 1997 ▸; García-Alvarez *et al.*, 2003 ▸) and to the position of the domain-swapped chain *A* CH1 and chain *B* CH2 domains in the previous open dystrophin structure (PDB entry 1dxx) (Fig. 2 ▸). Using this model, molecular replacement yielded a TFZ score of 46.1 and an LLG score of 4252.272. Initial refinement gave an *R*_work_ of 0.3315 and an *R*_free_ of 0.3978. To ensure that the *AlphaFold*2 model was not biasing refinement towards a closed state, we tested using chain *A* of PDB entry 1dxx as a molecular-replacement model. This yielded a TFZ score of 59.0, an LLG score of 2528.177 and an initial *R*_work_ of 0.5054 and *R*_free_ of 0.5526. Based on the refinement statistics, the closed-conformation *AlphaFold*2-derived molecular-replacement model was used for further refinement. This choice was supported by the presence of inter-chain 2*F*_o_ − *F*_c_ electron density for the linker region connecting CH1 and CH2 in all chains of the asymmetric unit (Fig. 2 ▸).

The structural model was refined to an *R*_work_ value of 0.2853 which did not decrease with further iterations. We suspected that the crystal exhibited twinning and used *phenix.xtriage* to evaluate the twinning models, finding that the model (*l*, −*k*, *h*) decreased the *R*_work_ to 0.2239. Further refinement and addition of waters reduced the *R*_work_ to its final reported value of 0.2157 and *R*_free_ to 0.2617. Structure-solution and refinement statistics are summarized in Table 1 ▸. The final model was deposited in the Research Collaboratory for Structural Bioinformatics Protein Data Bank (PDB) as PDB entry 9d58.

### 3.2. Structure analysis

**Figure 3:**
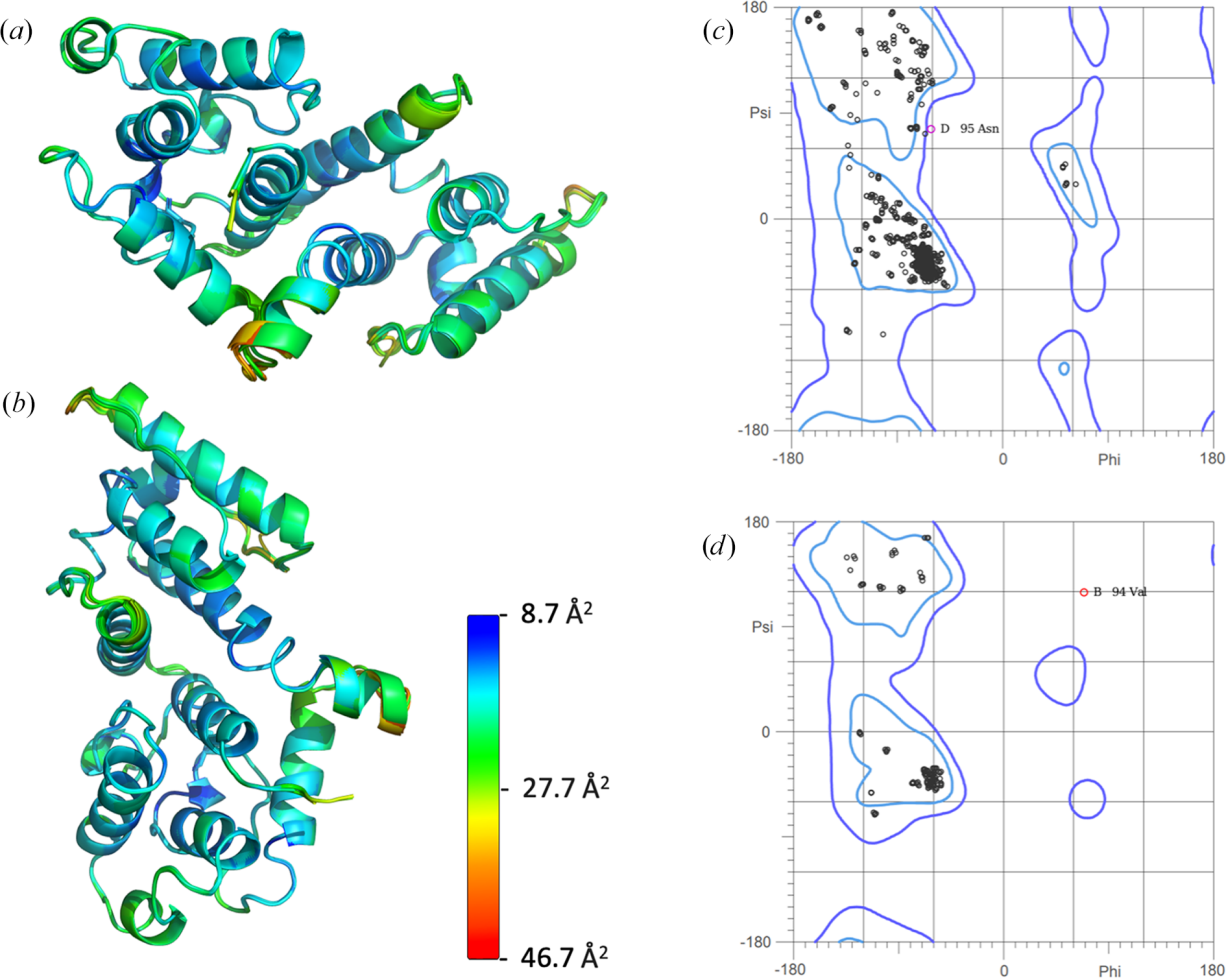
(*a*, *b*) *B* factors of the refined model PDB entry 9d58 for aligned chains *A*–*D* viewed from two orthogonal viewpoints. The *B* factor is scaled from low (cyan) to high (red). (*c*) General case Ramachandran plot for peptide backbone φ–ψ angles of all chains. (*d*) Isoleucine and valine Ramachandran plot of φ–ψ angles of all chains.

The structural model shows density for peptide backbone residues 13–244, with most residues exhibiting resolved density for side chains. The Ramachandran plot shows 97.60% of residues falling within the favored region, 2.18% in allowed regions and 0.22% as outliers (Fig. 3 ▸). The only Ramachandran outliers were residues Val94 in chain *B* and Asn95 in chain *D*. Val94 in chain *B* appears to be pushed into steric strain by Ile96, which is in a different rotamer conformation in chain *B* than in chains *A*, *C* and *D*. The electron density in this region supports this backbone position. The average overall *B* factor for the model is 19.13 Å^2^. The N- and C-termini exhibited the highest *B*-factor values (Fig. 3 ▸) and the loop between CH1 and CH2 (residues 130–135) had an average *B* factor of 33.79 Å^2^ as calculated using the *PyMOLaverage_b* script.

The closed-conformation structure is highly homologous to related proteins crystallized in a closed state (Fig. 2 ▸) and to the positions of the CH1 and CH2 domains in the open domain-swapped conformations of existing dystrophin ABD1 (PDB entry 1dxx) and utrophin ABD1 (PDB entry 1qag) structures. The peptide-backbone r.m.s.d. between our new structure PDB entry 9d58 and homologous tandem CH-domain proteins indicate that the closed conformation is highly similar (r.m.s.d. of <2 Å) to all homologs except T-fimbrin (Table 2 ▸). The r.m.s.d. between the individual CH domains is even smaller (<2 Å for CH1 and <2 Å for CH2 for all but filamin). The surface area buried by the interface between CH1 and CH2 (Table 3 ▸) is also similar (2500.6 Å^2^ for PDB entry 9d58) compared with the surface area of the CH1–CH2 domain interface of chains *A* and *B* in PDB entry 1dxx (2337.5 Å^2^), with many of the same side-chain interactions stabilizing the interface (Fig. 2 ▸).

In domain-swapped dystrophin and utrophin dimers, the linker Gln134–Asn137 in dystrophin forms a helix that is extended away from the CH1 and CH2 domains. In the closed conformation, the helix melts and the linker forms a turn. Electron density for each residue in this turn is resolved in PDB entry 9d58 in all four chains of the asymmetric unit (Fig. 2 ▸). The C-terminal residues of PDB entry 1dxx form a domain-swapped β-sheet, which presumably provides further stabilization of the extended open conformation. These C-terminal residues are not resolved in the electron density of the closed conformation seen in PDB entry 9d58.

**Figure 4:**
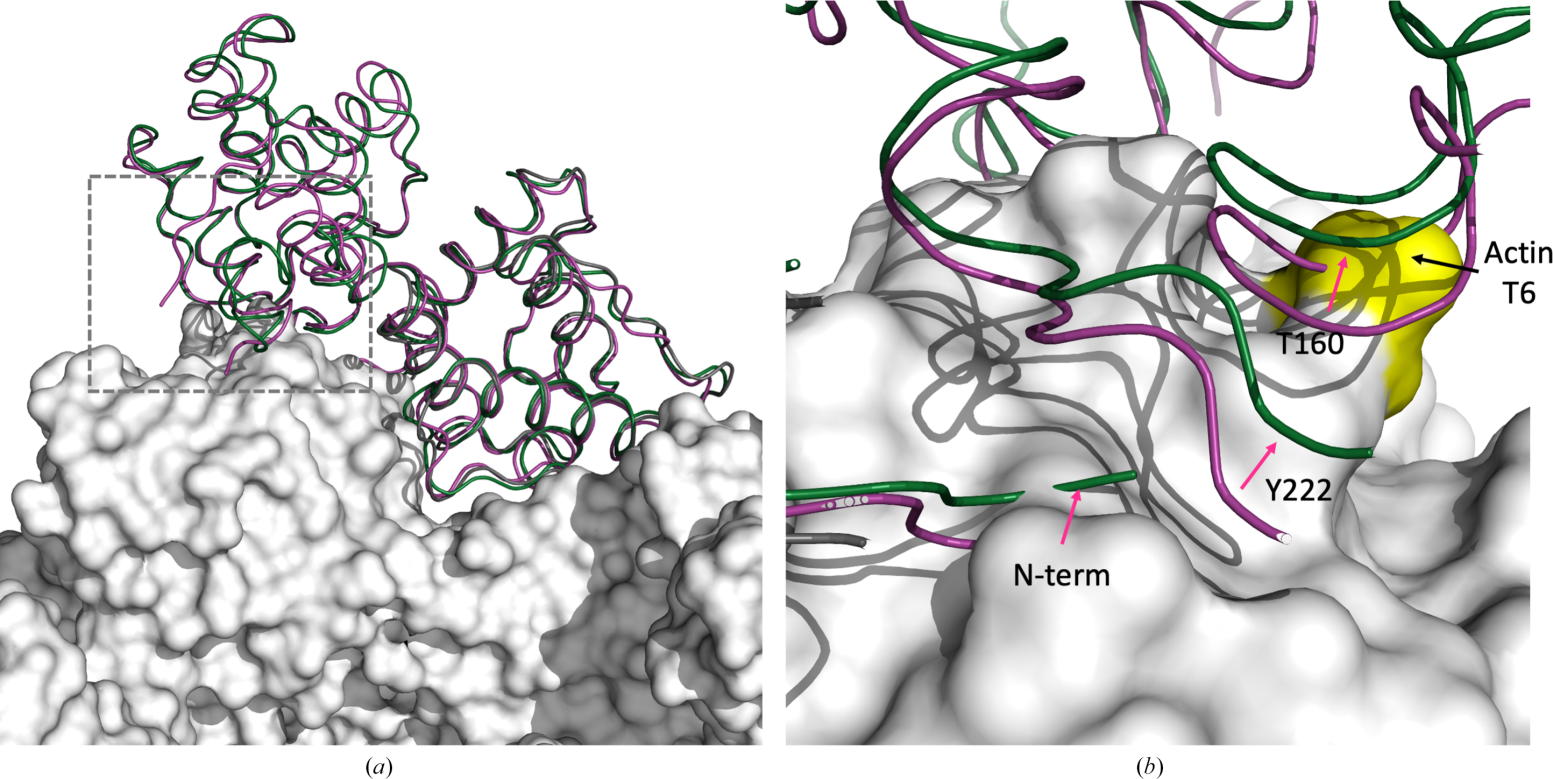
Actin-bound homology models. (*a*) PDB entry 9d58 (green) and PDB entry 1dxx (violet) aligned with the CH1 domain of utrophin bound to actin (PDB entry 6m5g). The box indicates the region of CH2 that clashes with actin in PDB entry 1dxx. (*b*) The inset box in (*a*) showing the position of loops in CH2 that are positioned further from the surface of actin. In the closed CH1–CH2 conformation, actin residue Thr6 clashes with dystrophin residues 157–162. The resolved N-terminal residues of PDB entries 9d58 and 1dxx also clash with actin in this model, indicating that structural changes occur in CH2 upon actin binding.

Alignment of individual CH1 domains of the closed-conformation states with a cryo-EM structure of utrophin CH1 bound to actin filaments (PDB entry 6m5g; Kumari *et al.*, 2020 ▸) reveals differences in the relative orientation of individual CH2 domains and supports the hypothesis that the CH1–CH2 interface ‘opens’ during or before actin binding (Fig. 4 ▸) as steric clashes between residues in CH2 with actin are present in the aligned model. Spectroscopic studies using double electron–electron resonance spectroscopy also support this hypothesis, suggesting that dystrophin ABD1 is in an equilibrium between open and closed conformations in solution (Lin *et al.*, 2012 ▸). Together, our new structure provides molecular details of the closed conformation that the dystrophin ABD1 monomer adopts in solution and confirms that the domain-wapped closed state seen for dystrophin ABD1 dimers in PDB entry 1dxx also occurs in a dystrophin monomer.

## Supplementary Material

PDB reference: human dystrophin tandem calponin homology actin-binding domain, 9d58

## Figures and Tables

**Table 1:** Data-collection and refinement statistics

Data collection	
X-ray source	SSRL beamline BL12-2
Wavelength (Å)	0.979
Data-collection temperature (K)	100
Detector	Dectris PILATUS 6M PAD
Exposure time (s)	0.5
Crystal-to-detector distance (mm)	300
Angle increment (°)	0.25
Resolution range (Å)	58.51–1.944 (1.978–1.944)
Space group	*P*12_1_1
*a*, *b*, *c* (Å)	59.774, 143.138, 59.862
α, β, γ (°)	90, 102.21, 90
Matthews coefficient (Å^3^ Da^−1^)	2.24
Solvent content (%)	45.2
Total reflections	218866
Unique reflections	70460 (7001)
Multiplicity	3.1
Mosaicity (°)	0.2
Completeness (%)	97.79 (97.88)
Mean *I*/σ(*I*)	7.07 (2.22)
Wilson *B* factor (Å^2^)	18.8
*R*_merge_	0.104 (0.480)
*R*_meas_	0.126 (0.580)
*R*_p.i.m._	0.069 (0.322)
CC_1/2_	0.995 (0.838)
Refinement statistics	
Reflections used in refinement	70460 (7001)
Reflections used for *R*_free_	3487 (319)
*R*_work_	0.2158 (0.2671)
*R*_free_	0.2619 (0.3168)
No. of non-H atoms	
Total	7478
Macromolecules	7239
Ligands	0
Solvent	239
No. of protein residues	926
R.m.s.d., bond lengths (Å)	0.004
R.m.s.d., angles (°)	0.64
Ramachandran favored (%)	97.6
Ramachandran allowed (%)	2.18
Ramachandran outliers (%)	0.22

**Table:** (*a*) Actin-binding domain alignment.

Protein name	PDB code, chain	Sequence†	Identical residues	Sequence identity (%)	R.m.s.d. (Å)
Dystrophin	9d58, *A*	23–209	NA	NA	NA
Dystrophin	1dxx, *A*	21–207	187	100	0.849
Utrophin	1qag, *A*	7–192	135	72.2	0.564
Plectin	1mb8, *A*	20–207	87	45.1	1.061
α-Actinin 1	2eyi, *A*	12–191	80	42.6	1.269
Filamin-B	2wa5, *A*	25–212	68	35.1	1.106
T-fimbrin	1aoa, *A*	29–247	49	22.2	2.249
Spectrin-β (L253P)	6anu, *G*	NA	NA	NA	NA

**Table:** (*b*) CH1 alignment.

Protein name	PDB code, chain	Sequence	Identical residues	Sequence identity (%)	R.m.s.d. (Å)
Dystrophin	9d58, *A*	23–117	NA	NA	NA
Dystrophin	1dxx, *A*	21–115	95	100	0.374
Utrophin	1qag, *A*	7–101	72	75.8	0.447
Plectin	1mb8, *A*	75–168	53	53.5	0.634
α-Actinin 1	2eyi, *A*	12–106	49	51.0	0.634
Filamin-B	2wa5, *A*	25–121	41	41.8	0.542
T-fimbrin	1aoa, *A*	29–135	28	26.2	1.441
Spectrin-β (L253P)	6anu, *G*	63–157	54	56.2	1.190

**Table:** (*c*) CH2 alignment.

Protein name	PDB code, chain	Sequence	Identical residues	Sequence identity (%)	R.m.s.d. (Å)
Dystrophin	9d58, *A*	142–209	NA	NA	NA
Dystrophin	1dxx, *A*	140–207	68	100	0.287
Utrophin	1qag, *A*	126–192	44	64.7	0.512
Plectin	1mb8, *A*	141–207	27	39.7	0.783
α-Actinin 1	2eyi, *A*	125–191	29	42.6	0.416
Filamin-B	2wa5, *A*	148–212	23	33.3	3.035
T-fimbrin	1aoa, *A*	173–247	17	22.1	0.722
Spectrin-β (L253P)	6anu, *G*	NA	NA	NA	NA

† Sequence numbering corresponds to the residues of the indicated chain.

**Table 3:** CH1–CH2 interface surface areas

Protein name	PDB code	Interface area (Å^2^)
Dystrophin	9d58	2500.6
Dystrophin	1dxx	2337.5
Utrophin	1qag	2182.5
Plectin	1mb8	2667.8
α-Actinin 1	2eyi	2504.7
Filamin-B	2wa5	2373.8
T-fimbrin	1aoa	1763.8
